# Impact of LbSapSal Vaccine in Canine Immunological and Parasitological Features before and after *Leishmania chagasi*-Challenge

**DOI:** 10.1371/journal.pone.0161169

**Published:** 2016-08-24

**Authors:** Lucilene Aparecida Resende, Rodrigo Dian de Oliveira Aguiar-Soares, Henrique Gama-Ker, Bruno Mendes Roatt, Ludmila Zanandreis de Mendonça, Marina Luiza Rodrigues Alves, Denise da Silveira-Lemos, Rodrigo Corrêa-Oliveira, Olindo Assis Martins-Filho, Márcio Sobreira Silva Araújo, Ricardo Toshio Fujiwara, Nelder Figueiredo Gontijo, Alexandre Barbosa Reis, Rodolfo Cordeiro Giunchetti

**Affiliations:** 1 Laboratório de Biologia das Interações Celulares, Departamento de Morfologia, Universidade Federal de Minas Gerais, Belo Horizonte, Minas Gerais, Brazil; 2 Laboratório de Imunopatologia, Núcleo de Pesquisas em Ciências Biológicas/NUPEB, Instituto de Ciências Exatas e Biológicas, Universidade Federal de Ouro Preto, Ouro Preto, Minas Gerais, Brazil; 3 Laboratório de Imunologia e Genômica de Parasitos – Departamento de Parasitologia, Instituto de Ciências Biológicas, Universidade Federal de Minas Gerais, Belo Horizonte, Minas Gerais, Brazil; 4 Laboratório de Imunologia Celular e Molecular, Centro de Pesquisa René Rachou, Fundação Oswaldo Cruz, Belo Horizonte, Minas Gerais, Brazil; 5 Laboratório de Biomarcadores de Diagnóstico e Monitoração, Centro de Pesquisa René Rachou, Fundação Oswaldo Cruz, Belo Horizonte, Minas Gerais, Brazil; 6 Laboratório de Fisiologia de Insetos Hematófagos, Departamento de Parasitologia, Instituto de Ciências Biológicas, Universidade Federal de Minas Gerais, Belo Horizonte, Brazil; Pasteur Institute of Iran, ISLAMIC REPUBLIC OF IRAN

## Abstract

Dogs represent the most important domestic reservoir of *L*. *chagasi* (syn. *L*. *infantum*). A vaccine against canine visceral leishmaniasis (CVL) would be an important tool for decreasing the anxiety related to possible *L*. *chagasi* infection and for controlling human visceral leishmaniasis (VL). Because the sand fly salivary proteins are potent immunogens obligatorily co-deposited during transmission of *Leishmania* parasites, their inclusion in an anti-*Leishmania* vaccine has been investigated in past decades. We investigated the immunogenicity of the “LbSapSal” vaccine (*L*. *braziliensis* antigens, saponin as adjuvant, and *Lutzomyia longipalpis* salivary gland extract) in dogs at baseline (T_0_), during the post-vaccination protocol (T_3rd_) and after early (T_90_) and late (T_885_) times following *L*. *chagasi*-challenge. Our major data indicated that immunization with “LbSapSal” is able to induce biomarkers characterized by enhanced amounts of type I (tumor necrosis factor [TNF]-α, interleukin [IL]-12, interferon [IFN]-γ) cytokines and reduction in type II cytokines (IL-4 and TGF-β), even after experimental challenge. The establishment of a prominent pro-inflammatory immune response after “LbSapSal” immunization supported the increased levels of nitric oxide production, favoring a reduction in spleen parasitism (78.9%) and indicating long-lasting protection against *L*. *chagasi* infection. In conclusion, these results confirmed the hypothesis that the “LbSapSal” vaccination is a potential tool to control the *Leishmania chagasi* infection.

## Introduction

*Leishmania infantum* (syn. *L*. *chagasi*) is an intracellular protozoon that cause a severe systemic disease named visceral leishmaniasis (VL) [1]. It is widely distributed in the Mediterranean Basin, Middle East, and South America. VL has an annual incidence of approximately 500,000 cases [2]. Brazil declared a total of 50,060 clinical VL cases between 1990 and 2006, and this number accounts for 90% of all reported VL cases in the Americas; however, it is subject to substantial under-reporting [3].

Different geographical regions of the globe where visceral leishmaniasis is endemic, present the dogs as main reservoir for the *L*. *chagasi* that play a relevant role in transmission of the parasite [4,5]. Dogs are also excellent models for the study of VL because the natural history of the disease in dogs and in humans is similar [6], especially regarding parasite–host interaction, immune response, and the development of new vaccines [7]. The natural history of canine VL (CVL) has been well-described, particularly in regard to the parasite load in different tissues and the immunopathological changes according to progression of clinical forms [8,9,10,11,12,13,14,15,16,17].

A vaccine against CVL would be an important tool in the control of VL and would decrease the anxiety associated with *L*. *chagasi* infection in humans [18]. The induction of long-lasting cell-mediated immune response triggering high levels of protection against CVL is considered as prerequisite of an ideal vaccine for controlling the *L*. *chagasi* transmission. Different vaccine candidates against CVL have been reported showing the ability to induce immunoprotective mechanisms [19,20,21,22,23,24,25,26,27].

In this sense, it has been shown that Leishmune^®^ (fucose manose ligand plus saponin as adjuvant; Zoetis, São Paulo, Brazil) presented 95% protection in a phase III study [28]. Leishmune^®^ provided protection associated with the ability to trigger early and persistent activation of neutrophils and monocytes, in addition to activation of T CD4^+^ and T CD8^+^ lymphocytes displaying high levels of IFN-γ in the CD4^+^ T-cell subset [29,30]. Additionally, the Leish-Tec^®^ vaccine (Hertape Calier, Juatuba, Brazil; A2 antigen plus saponin as adjuvant) has also been shown to induce increased levels of IFN-γ in four out of seven dogs with *L*. *chagasi-*infected bone marrow [21]. More recently, a vaccine composed of excreted/secreted antigens of *L*. *infantum* promastigotes, referred as LiESAp has been shown to induce increased levels of IFN-γ and nitric oxide (NO), thus supporting its leishmanicidal effect [31]. The efficacy of the LiESAp vaccine was reported as 92% in a double-blind randomized study [19]. LiESAp is commercially available as CaniLeish^®^ (Virbac, Carros, France) and is able to induce a Th1 profile and reduce the parasitic load of infected macrophages co-cultured with lymphocytes from immunized dogs [32].

The LbSap (*L*. *braziliensis* crude antigens plus saponin as adjuvant) and “LbSapSal” (*L*. *braziliensis* crude antigens plus saponin as adjuvant and *Lutzomyia longipalpis* saliva) vaccines against CVL has also shown to induce a strong immune-mediated response. The LbSap and “LbSapSal” presented higher levels of circulating T-cell subsets (CD4^+^, CD8^+^) and B lymphocytes (CD21^+^), as well as *Leishmania*-specific CD8^+^ and CD4^+^ T cells [20,22,25,27]. Furthermore, LBSap-vaccinated dogs presented high IFN-γ and low interleukin (IL)-10 and transforming growth factor (TGF)-β1 expression in the spleen, resulting in a significant reduction of parasite load in this tissue [25]. Additionally, LbSap has been shown to induce a prominent pro-inflammatory immune response characterized by increased levels of both IL-12 and IFN-γ and decreased levels of TGF-β by peripheral blood mononuclear cells (PBMCs), which were associated with parasite control in dogs [26].

The incorporation of salivary proteins of sand flies have been widely used in experimental challenge studies, and the results suggest that this could be a good strategy to protect against *Leishmania* infection [22,33,34,35,36,37,38,39,40,41]. Previous studies of dogs using the “LbSapSal” vaccine displayed higher counts of circulating and *Leishmania*-specific CD8^+^ T cells in addition to high nitric oxide (NO) production [22] and reduction of splenic parasite load [27]. Considering the inclusion of saliva from sand flies as a promising compound in vaccines against *Leishmania* infection, we describe additional biomarkers induced by the “LbSapSal” vaccine in dogs. We considered the previous vaccine protocol (T_0_), post-vaccine protocol (T_3rd_), and early (T_90_) and late (T_885_) periods of the *L*. *chagasi*-challenge.

## Materials and Methods

### Ethics statement

All research involving dogs was carried out according to the regulations of the Brazilian Society of Science in Animal Research, adopted by the Federal University of Ouro Preto Ethics Committee in Animal Experimentation, that approved the technical procedures using dogs under protocol number 2010/71.

### Animals, vaccination, and experimental challenge with *Leishmania chagasi* plus *Lutzomyia longipalpis* saliva

All dogs included in this study, were born and bred in the kennel facility at Universidade Federal de Ouro Preto in Ouro Preto, Minas Gerais, Brazil. This kennel facility was established with male and female mongrel dogs, with negative serological and molecular/parasitological diagnosis for *Leishmania* infection, donated by the local zoonosis control center from Belo Horizonte, Minas Gerais, Brazil. The filial generations, comprising of offsprings resulting from the cross between the original mongrel dogs pairs were used further maintain the mongrel dog colony. In the present study, twenty mongrel dogs (10 males and 10 females), with seven months of age and negative results for indirect fluorescence immunoassay to anti-*Leishmania* antibodies, were selected and received an anti-helmintic treatment and were submitted to poly-vaccination protocols, including rabies (Tecpar, Curitiba-PR, Brazil), leptospira, parvovirus, canine distemper, type II adenovirus, parainfluenza and coronavirus (Vanguard^®^ HTLP 5/CV-L; Pfizer Animal Health, New York, NY, USA). Prior the experimental onset, the animals were maintained in quarantine in kennel runs (4m length x 2m width x 3m height) completely covered with stainless steel wire mesh to prevent the entry of sand flies. The kennel facilities were sprayed with pyrethroid insecticide, every three months, as a measure to control insect access. Each run was installed with an infrared lamp heating (250 watts) to ensure the thermal comfort of animals during the night and on cold days. All the dogs were housed without environmental enrichment (e.g. toys, exercise regimes, etc). The dogs were monitored twice a day, as routine inspection, during which the responsible veterinary was in charge of stimulating and playing with them to aiming to avoid behavior problems and minimize their suffering or distress throughout the study. All the experimental procedures described in this study, including this aspect of animal care was approved by Federal University of Ouro Preto Ethics Committee in Animal Experimentation (protocol number 2010/71). The animals were maintained with water and food *ad libitum* throughout the experiment time. The euthanasia of all dogs was performed under the supervision of a veterinarian physician, using barbiturate anesthetic (Thiopental Sodium, 35mg/kg, iv) followed by intravenous injection of saturated solution of potassium chloride.

Four groups of dogs involved in experiments were treated as follows: (i) “Control” (C) group (n = 5; subcutaneous injections of 1mL of sterile 0.9% saline); (ii) “Sal” group (n = 5; subcutaneous injections of salivary *Lutzomyia longipalpis* gland extract (SGE; obtained as previously described–[22]) in 1mL of sterile 0.9% saline); (iii) “LbSal” group (n = 5; subcutaneous injections of 600μg of *L*. *braziliensis* promastigote antigen and SGE in 1mL of sterile 0.9% saline); and (iv) “LbSapSal” group (n = 5; subcutaneous injections of 600μg of *L*. *braziliensis* promastigote antigen plus 1mg of saponin and SGE in 1mL of sterile 0.9% saline). All animals received three injections in the right flank at 28days intervals. The *L*. *chagasi*-experimental challenge in all dogs was performed after 100 days of vaccination protocol, using intradermal 10^7^ promastigotes during the stationary phase of cultivation. The challenge was performed in the inner side of the left ear including five *Lutzomyia longipalpis* salivary gland acini. All the analyzed dogs were euthanized 885 days after *L*. *chagasi*-experimental challenge and the spleens were collected to evaluate parasite loads. The rationale for choosing such a long endpoint (885 days after challenge) was based on the chronic course of the experimental canine visceral leishmaniasis after intradermal *L*. *chagasi*-challenge as previously reported [27].

### Vaccine preparation

The vaccine was obtained as previously described [22]. Briefly, *L*. *braziliensis* (MHOM/BR/75/M2903) promastigotes were obtained by *in vitro* culture in Neal, Novy, Nicolle/Liver Infusion Triptose media [20], and the parasite was fully disrupted by ultrasound treatment (40W, 1min, 4°C), aliquoted and stored at −80°C. Protein concentration was determined according to the method of Lowry [42]. The SGE used in this study as the antigenic component of the vaccine was obtained from *Lutzomyia longipalpis* females that were not fed, aged 4 days, and dissected in slightly hypotonic unbuffered saline 0.8%, as described in [22]. After collection, the glands were disrupted in a sonicator for 10 seconds and centrifuged at 10,000*g* for 2min. The supernatant was collected and stored in a freezer at -80°C until use.

### Blood sample collection and PBMCs culture *in vitro*

Jugular vein was used for collecting 20mL of peripheral blood in sterile heparinized syringes. We analyze distinct times: baseline before vaccination (T_0_), 15 days after third immunization dose (T_3rd_) as well as early (90 days—T_90_) and late (885 days—T_885_) after experimental *L*. *chagasi*-challenge. The PBMCs were isolated as previously described [20]. Briefly, the whole blood samples were added over 10mL of Ficoll-Hypaque (Histopaque^®^ 1077; Sigma, USA), centrifuged at 450*g* for 40 min at room temperature, and the PBMCs were washed twice with RPMI 1640 (450*g* for 10 min at room temperature). The PBMCs were resuspended in RPMI 1640 at 10^7^ cells/mL. The PBMCs cultures were performed in 48-well flat-bottom tissue culture plates (Costar, Cambridge, MA, USA). The *in vitro* assays were performed using 50μL of PBMCs (5.0×10^5^ cells/well) with 100μL of *vaccine L*. *braziliensis-solubl*e antigen (VSA; 25μg/mL) or 100μL of soluble *L*. *chagasi* antigen (SLcA; 25 μg/ml). The control cultures (CC; unstimulated) were analyzed using 100μL of RPMI in place of the antigenic stimulus. Incubation was performed in a humidified 5% CO_2_ atmosphere at 37°C for 5 days, after which time the supernatants were collected and stored in a freezer at −80°C for detection of cytokine and NO.

### Quantification of cytokines

The quantification of cytokines was carried out by enzyme-linked immunosorbent assay (ELISA) according to the manufacturer’s instructions (R&D Systems, Minneapolis, MN, USA), as previously described [26]. Minimum cytokine sensitivity detection levels were 62pg/mL (IL-12), 63pg/mL (TNF-α and IFN-γ), 78pg/mL (IL-4 and IL-10) and 31pg/mL (TGF-β). The analysis of IL-12 (anti-canine IL-12/IL-23 p40, catalog number DY1969), IFN-γ (anti-canine IFN-γ, catalog number DY781B), tumor necrosis factor (TNF)-α (anti-canine TNF-α/TNFSF1A immunoassay; catalog number DY1507) and IL-10 (anti-canine IL-10, catalog number DY735) cytokines were performed using DuoSet ELISA. Quantikine^®^ kit (mouse/rat/porcine/canine TGF-β1 immunoassay, catalog number MB100B) (R&D Systems, Minneapolis, MN, USA) was used to measure TGF-β levels. The analysis of IL-4 levels employed the capture antibody (monoclonal anti-canine IL-4 antibody—catalog number MAB7541); the standard curve was obtained using recombinant canine IL-4 (catalog number 754CL); and streptavidin (DY998; R&D Systems/USA), biotinylated anti-canine IL-4 antibody (catalog number: BAF754) and substrate solution (1:1 mixture of H_2_O_2_ and tetramethylbenzidine; product code 50-76-4) were used.

All experiments were performed according to the instructions of R&D Systems using 96-well plates (Corning Incorporated, COSTAR^®^, Washington, DC, USA). The microplate automatic reader (EL800; Biotek, Winosski, VT, USA) was employed at a wavelength of 450nm.

### NO production

The measurement of NO levels in supernatants of PBMCs cultures was performed by indirect method that quantify the nitrite concentration by Griess reaction [43,44]. Briefly, 100μL of Griess reagent (1% sulfanylamide, 0.1% naphthylethylene-diamide-dihydrochloride, and 2.5% phosphoric acid; all from Sigma, USA) was mixed with 100μL aliquot of cell-free culture supernatant. The microplate reader (Biotek, EL800) was used to analyze the absorbance at 540nm, after 10 min of incubation at room temperature in the dark.

The final nitrite concentration was determined based on a standard curve interpolation constructed by using sodium nitrite solutions in the range of 0–100μM. The interference of nitrites already present in the culture medium was discounted; data were calculated by taking into account the blank, as control reaction, assayed by using the PBMCs cultures medium. The data were expressed as nitrite concentration (μM).

### Spleen samples and parasite load measurement by qPCR

The spleen specimens (5mm) were collected during necropsy and stored at -80°C until use for DNA extraction. The Wizard^™^ Genomic DNA Purification Kit (Promega, Madison, WI, USA) was used to extract total genomic DNA in 20mg of spleen following manufacturer’s recommendations.

Primers that amplified a 90-bp fragment of a single copy of the DNA polymerase gene of *L*. *chagasi* were used to analyze the spleen parasite burden by quantitative polymerase chain reaction (qPCR). For the q PCR analysis 200nM forward and reverse primers, 5μL of template DNA and 16SYBER GREEN reaction master mix (Applied Biosystems, Grand Island, NY, USA) were used in a final volume of 25μL. As previously described [45], we used the targets the DNA polymerase gene *L*. *chagasi* (GenBank accession: AF009147) and the pair of primers (forward: 5’ TGT CGC TTG CAG ACC AGA TG 3’; reverse: 5’ GCA TCG CAG GTG TGA GCA C 3’). The PCR reactions employed an initial denaturation at 95°C (10 min), 40 denaturation cycles at 95°C (15 seconds), in addition to annealing and extension at 60°C (1min.). The pGEMH T plasmids (Promega) was used for constructing stardart curves, for each run, containing inserts of interest [46]. The GAPDH gene (115-bp fragment GenBank accession number AB038240) was used to analyze the integrity of the samples. The primers 5’TTCCACGGCACAGTCAAG 3’ (forward) and 5’ ACTCAGCACCAGCATCAC 3’ (reverse) were used for GAPDH gene amplification. The spleen parasite load was performed in duplicate and calculated by interpolation from the standard curve included in the same experimental batch. The data was expressed as number of *L*. *chagasi* organisms/20ng of total DNA.

### Statistical analysis

The Prism 5.0 software package (Prism Software, Irvine, CA, USA) was used to evaluate data distribution normality by Kolmogorov-Smirnoff test and for further statistical analyses. One-way analysis of variance (ANOVA) followed by Tukey's multiple comparison were used for comparisons of cytokine profiles, NO levels and parasite load amongst the experimental groups. Student’s t-test was used for intra-group comparisons between the control cultures (CC) and antigen-stimulated cultures (VSA or SLcA-stimuli *in vitro*). Pearson correlation analysis was further applied to evaluate the relationship between spleen parasite burden and NO profiles. Additionally, the cytokine networks were assembled using Cytoscape software version 2.8.2 (Institute of Systems Biology, Seattle, USA), for each experimental group (“Control”, “Sal”, “LbSal”, and LBSapSal) in all times analyzed (T_0_, T_3rd_, T_90_, and T_885_), based on the correlations indices obtained by Pearson correlation analysis. The network constructed using distinct edges between nodes to identify negative or positive correlations, referred as moderate (0.37<r>0.67) or strong (r>0.68). In all cases, the significance were considered at *P*<0.05.

## Results

### “LbSapSal” immunization induced increased levels of IFN-γ before and after experimental *L*. *chagasi*-challenge

Aiming to verify whether the immunization protocols were able to induce the production of pro-inflammatory cytokines, we evaluated the profiles of TNF-α, IFN-γ, and IL-12 in CC or upon VSA or SLcA-stimuli *in vitro*.

**Fig 1:**
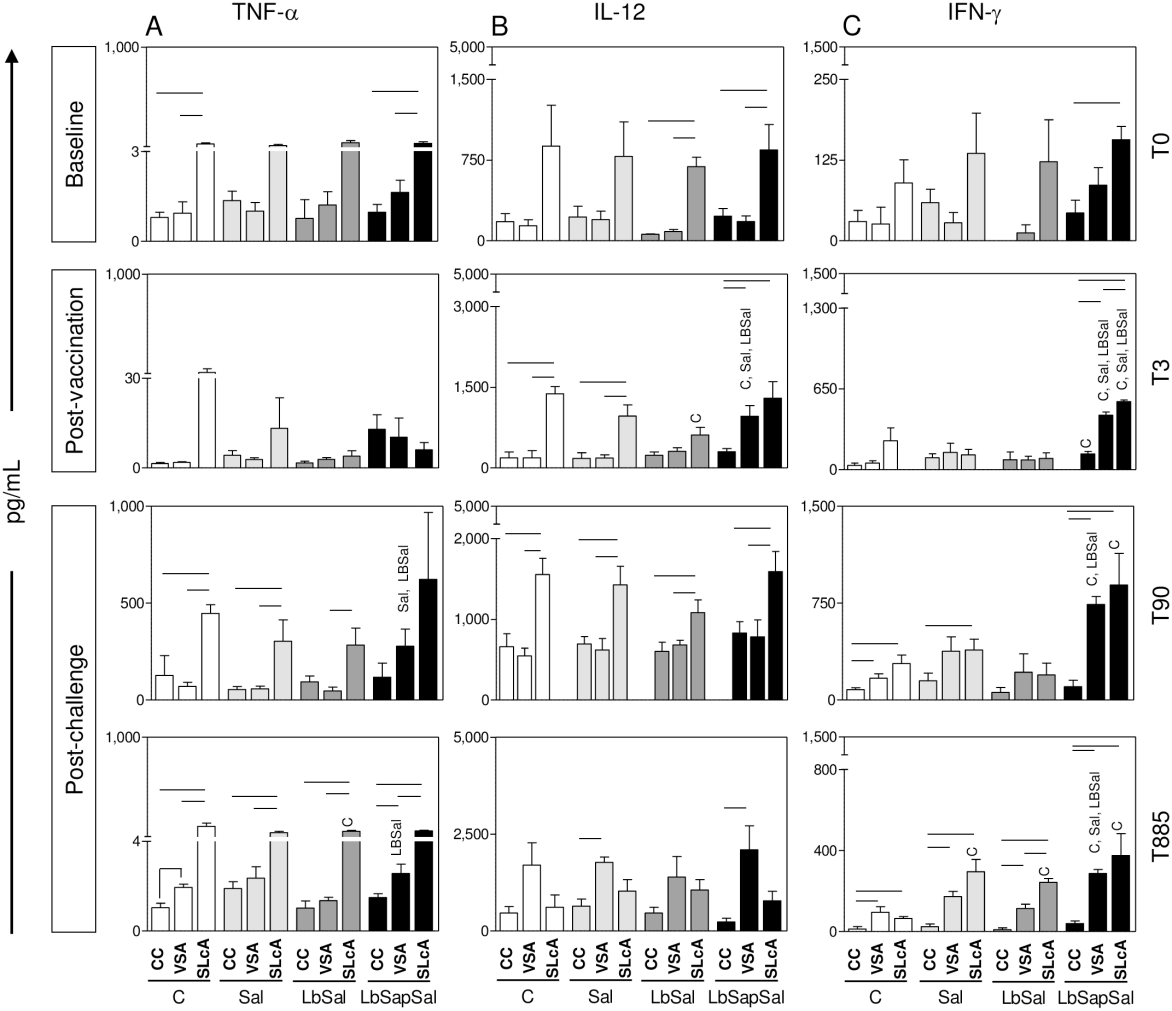
Impact of distinct immunization protocols on pro-inflammatory cytokine production. The levels of pro-inflammatory cytokine levels were measured in supernatants from PBMCs cultures maintained upon vaccine-soluble antigen (VSA) or soluble *Leishmania chagasi* antigen (SLcA) stimuli *in vitro*. Data were analyzed at baseline before vaccination (T_0_), 15 days after third immunization dose (T_3rd_) as well as early (90 days—T_90_) and late (885 days—T_885_) after experimental *L*. *chagasi*-challenge. The groups are represented as follows: C (“Control”; white bars); “Sal” (*Lutzomyia longipalpis* salivary glands; *light gray bars*); “LbSal” (antigen of *L*. *braziliensis* plus *Lutzomyia longipalpis* salivary glands; *dark gray bars*); and “LbSapSal” (*L*. *braziliensis* antigen plus saponin and *Lutzomyia longipalpis* salivary glands; black bars). The x-axis displays the different experimental groups (“Control”, “Sal”, “LbSal”, and “LbSapSal”) according to the *in vitro* stimuli (control culture [CC], VSA or SLcA). The y-axis represents the cytokine levels (pg/mL) for TNF-α (A), IL-12 (B) and IFN-γ (C). Data are presented as mean values ± standard deviations. The connecting lines represent significant difference (*P <0*.*05*) between the CC, VSA or SLcA-stimulated cultures. The symbols C, Sal and LbSal indicate significant differences in comparison to the “Control”, “Sal” and “LbSal” groups, respectively.

No significant differences were observed amongst the experimental groups at baseline (T_0_) (Fig 1A, 1B and 1C, upper panel).

Data analysis performed at (T_3rd_) post-vaccination did not show any differences in the TNF-α levels amongst the experimental groups, regardless the culture conditions. Analysis of IL-12 demonstrated that the “LbSapSal” group displayed increased levels (*P*<0.05) upon VSA-stimulation as compared with the same cultures in the “Control”, “Sal”, and “LbSal” groups (Fig 1B, middle panel). Moreover, the “LbSal” group showed reduced IL-12 levels (*P*<0.05) upon SLcA-stimulation as compared with the “Control” group (Fig 1B, middle panel). Interestingly, the analysis of IFN-γ showed that “LbSapSal” group presented increased levels (*P*<0.05) in CC as compared to the “Control” group (Fig 1C, middle panel). Additionally, “LbSapSal” group displayed increased IFN-γ levels (*P*<0.05) upon VSA and SLcA-stimuli in comparison with “Control”, “Sal”, and “LbSal” groups (Fig 1C, middle panel).

Analysis of the cytokine profile early after *L*. *chagasi*-challenge (T_90)_ demonstrated that “LbSapSal” group showed a significant increase of TNF-α levels (*P*<0.05) upon VSA-stimulation as compared to “Sal” and “LbSal” groups (Fig 1A, middle panel). No differences in the IL-12 levels were observed amongst the experimental groups, regardless the culture conditions. Analysis of IFN-γ, “LbSapSal” group displayed increased levels (*P*<0.05) upon VSA-stimulation as compared to “Control” and “LbSal” groups at T_90_ (Fig 1C, middle panel). Additionally, “LbSapSal” group showed a significant increase of IFN-γ (*P*<0.05) upon SLcA-stimulation as compared with the “Control” group (Fig 1C, middle panel).

Data analysis late after *L*. *chagasi*-challenge (T_885_) demonstrated that the “LbSal” group displayed increased TNF-α levels (*P*<0.05) upon SLcA-stimulation as compared with the “Control” group (Fig 1A, bottom panel). In addition, the “LbSapSal” group displayed higher levels of TNF-α (*P*<0.05) upon VSA-stimulation as compared with “LbSal” group (Fig 1A, bottom panel). No differences in the IL-12 levels were observed amongst the experimental groups, regardless the culture conditions. Interestingly, the analysis of IFN-γ revealed that “LbSapSal” group showed higher levels (*P*<0.05) upon VSA-stimulation as compared with the “Control”, “Sal” and “LbSal” groups (Fig 1C, bottom panel).

### “LbSapSal” induced lower levels of IL-4 and TGF-β but while unvaccinated dogs presented higher amounts of IL-10 after *L*. *chagasi*-challenge

Aiming to evaluate whether the immunization protocols would induce regulatory/anti-inflammatory cytokines, we further quantified the levels of IL-10, IL-4, and TGF-β upon VSA or SLcA-stimuli *in vitro*.

**Fig 2:**
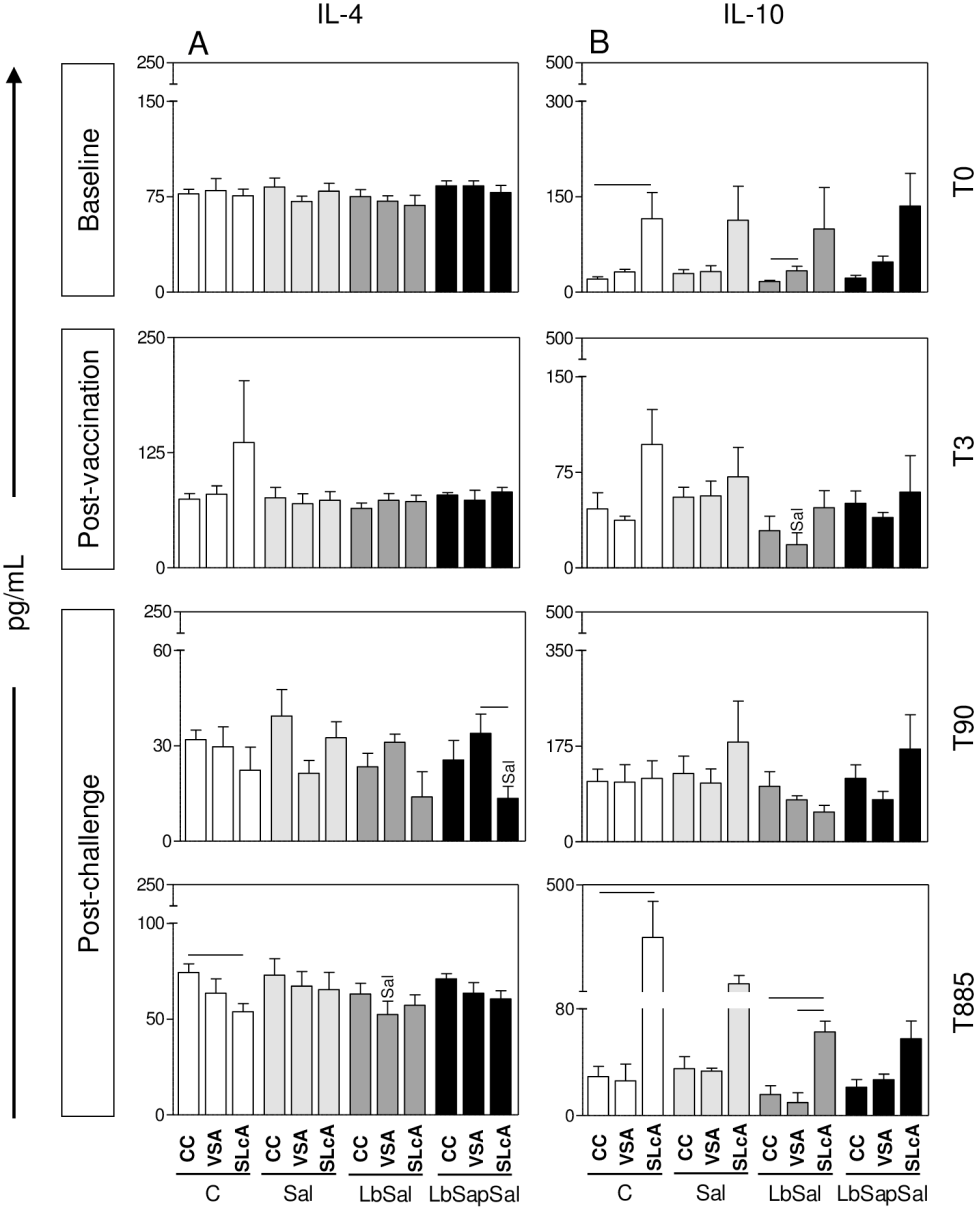
Impact of distinct immunization protocols on regulatory/anti-inflammatory cytokine production. The levels of regulatory/anti-inflammatory cytokines were measured in supernatants from PBMCs cultures maintained upon vaccine-soluble antigen (VSA) or soluble *Leishmania chagasi* antigen (SLcA) stimuli *in vitro*. Data were analyzed at baseline before vaccination (T_0_), 15 days after third immunization dose (T_3rd_) as well as early (90 days—T_90_) and late (885 days—T_885_) after experimental *L*. *chagasi*-challenge.The groups are represented as follows: C (“Control”; white bars); “Sal” (*Lutzomyia longipalpis* salivary glands; *light gray bars*); “LbSal” (antigen of *L*. *braziliensis* plus *Lutzomyia longipalpis* salivary glands; *dark gray bars*); and “LbSapSal” (*L*. *braziliensis* antigen plus saponin and *Lutzomyia longipalpis* salivary glands; black bars). The x-axis displays the different experimental groups (“Control”, “Sal”, “LbSal”, and “LbSapSal”) according to the *in vitro* stimuli (control culture [CC], VSA or SLcA). The y-axis represents the cytokine levels (pg/mL) for IL-4 (A) and IL-10 (B). Data are presented as mean values ± standard deviations. The connecting lines represent significant difference (*P <0*.*05*) between the CC, VSA or SLcA-stimulated cultures. The symbol Sal indicates significant differences in comparison to the “Sal” group.

No significant differences were observed amongst the experimental groups at baseline (T_0_) (Fig 2B, upper panel).

The results observed at the post-vaccination period (T_3rd_) demonstrated that the “LbSal” group showed a significant reduction in the IL-10 levels *(P*<0.05) upon VSA-stimulation as compared to the “Sal” group (Fig 2B, middle panel). No differences in IL-4 and TGF-β levels were observed amongst the experimental groups, regardless the culture conditions.

**Fig 3:**
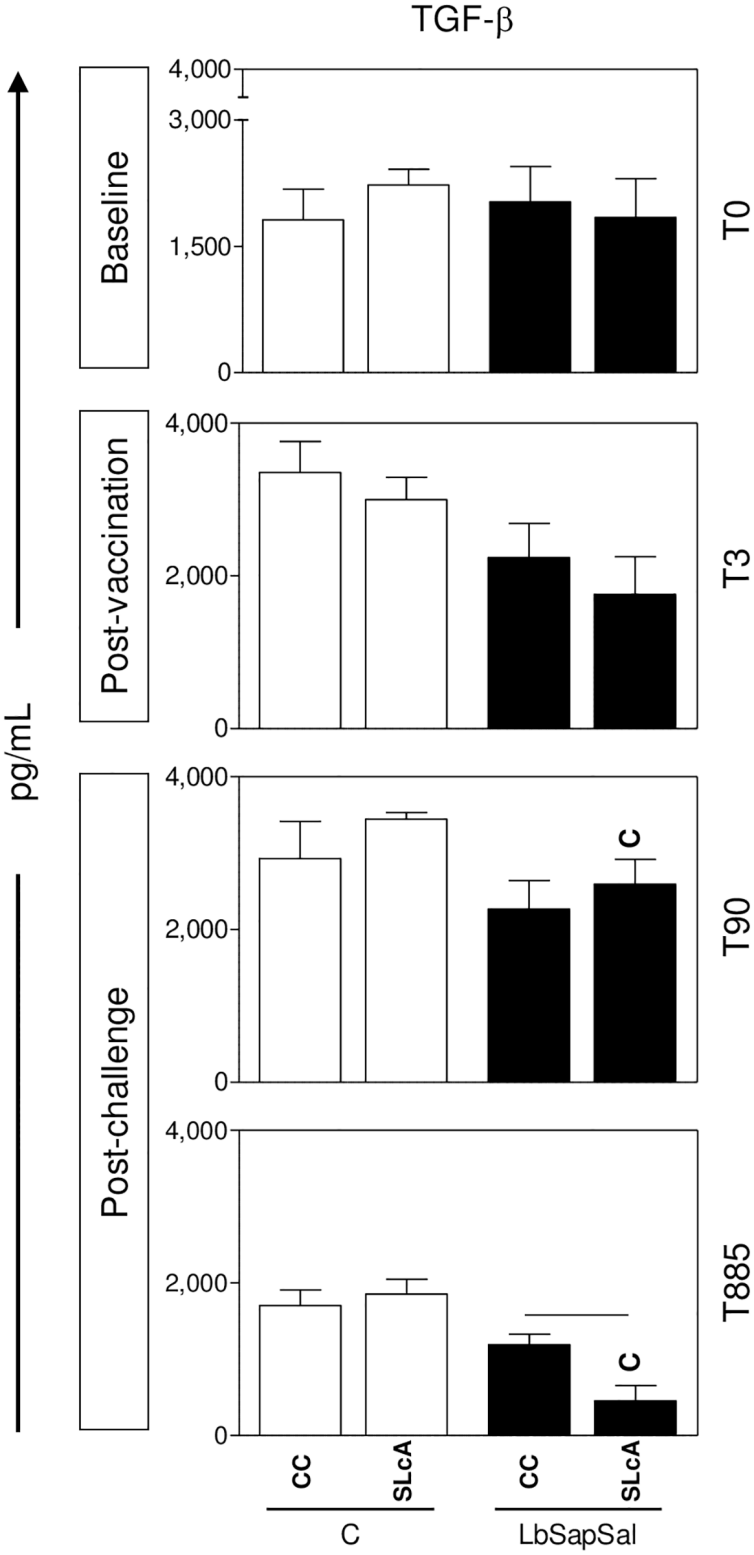
Impact of distinct immunization protocols on TGF-β production. The levels of TGF-β were measured in supernatants from PBMCs cultures maintained upon vaccine-soluble antigen (VSA) or soluble *Leishmania chagasi* antigen (SLcA) stimuli *in vitro*. Data were analyzed at baseline before vaccination (T_0_), 15 days after third immunization dose (T_3rd_) as well as early (90 days—T_90_) and late (885 days—T_885_) after experimental *L*. *chagasi*-challenge. The groups are represented as follows: C (“Control”; white bars) and “LbSapSal” (*L*. *braziliensis* antigen plus saponin and *Lutzomyia longipalpis* salivary glands; black bars).The x-axis displays the different experimental groups (“Control” and “LbSapSal”) according to the *in vitro* stimuli (control culture [CC] and SLcA). The y-axis represents the TGF-β levels (pg/mL). Data are presented as mean values ± standard deviations. The connecting lines represent significant difference (*P <0*.*05*) between the CC or SLcA-stimulated cultures. The symbol C indicates significant differences in comparison to the “Control” group.

Data mining performed early after *L*. *chagasi*-challenge (T_90_) revealed no differences in the IL-10 production amongst the experimental groups, regardless the culture conditions. However, “LbSapSal” group showed a significant reduction of IL-4 levels (*P*<0.05) upon SLcA-stimulation as compared to the “Sal” group (Fig 2A, middle panel). Interestingly, “LbSapSal” group also presented a significant reduction of TGF-β levels (*P*<0.05) upon SLcA-stimulation as compared the “Control” group (Fig 3, middle panel).

Analysis carried out late after *L*. *chagasi*-challenge (T_885_) did not differences in IL-10 levels amongst the experimental groups, regardless the culture conditions. The results showed that the “LbSal” group presented a significant reduction in the IL-4 levels (*P*<0.05) upon VSA-stimulation as compared with the “Sal” group (Fig 2B, bottom panel). Interestingly, “LbSapSal” group displayed a significant reduction of TGF-β levels (*P*<0.05) upon SLcA-stimulation as compared with the “Control” group (Fig 3, bottom panel).

### The hallmark of the cytokine network in the “LbSapSal” group is a balanced immune response regarding positive correlation between pro-inflammatory cytokines (IL-12, IFN-γ, TNF-α) and regulatory cytokines (IL-10)

**Fig 4:**
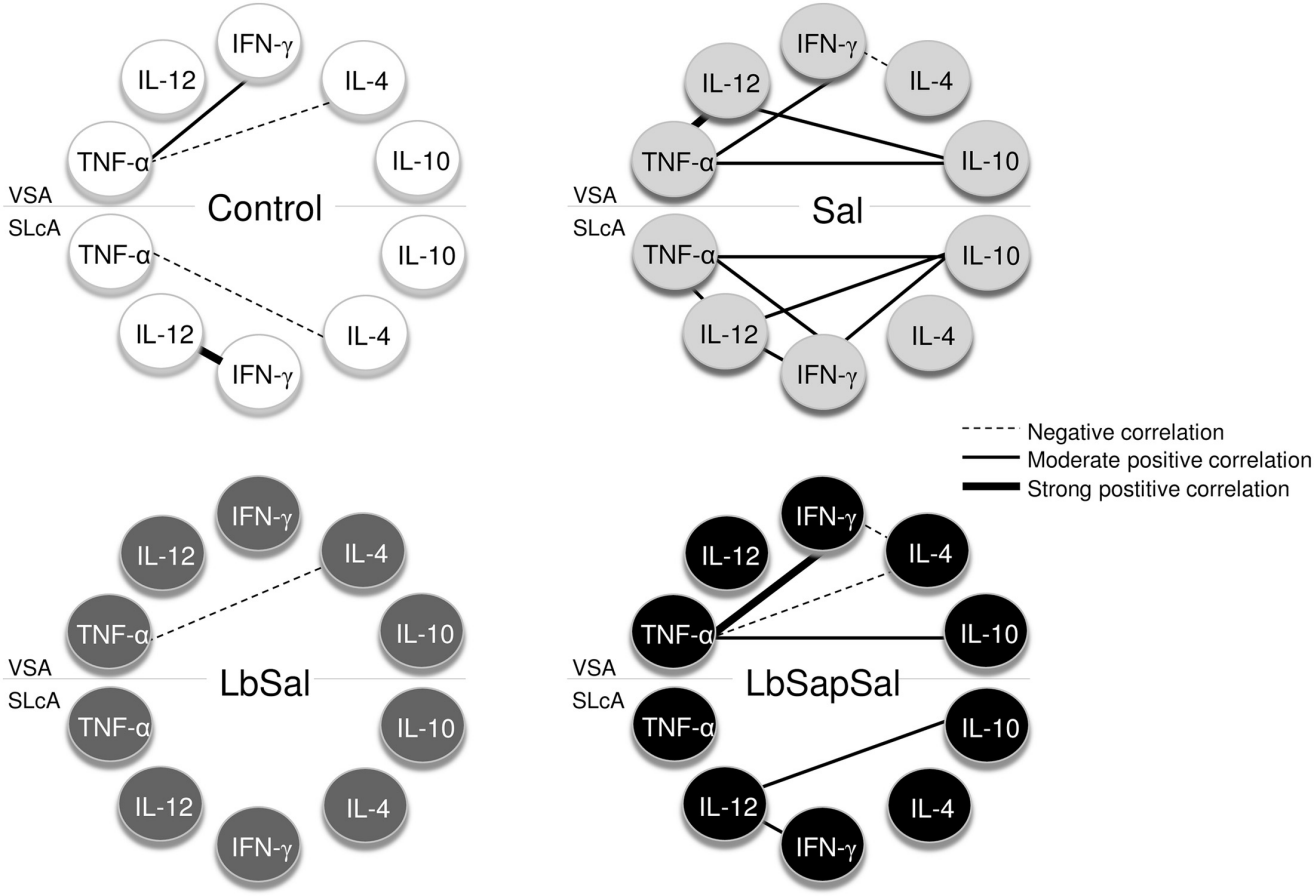
Biomarker networks triggered by distinct immunization protocols. Network correlation analysis were assembled for pro-inflammatory and regulatory cytokines measured in supernatants from PBMCs cultures maintained upon vaccine-soluble antigen (VSA) or soluble *Leishmania chagasi* antigen (SLcA) stimuli *in vitro*. Data were analyzed at baseline before vaccination (T_0_), 15 days after third immunization dose (T_3rd_) as well as early (90 days—T_90_) and late (885 days—T_885_) after experimental *L*. *chagasi*-challenge. The groups are represented as follows: C (“Control”; white nodes); “Sal” (*Lutzomyia longipalpis* salivary glands; light gray nodes); “LbSal” (*L*. *braziliensis* antigen plus *Lutzomyia longipalpis* salivary glands; dark gray nodes) and “LbSapSal” (*L*. *braziliensis* antigen plus saponin and *Lutzomyia longipalpis* salivary glands; black nodes). Each connecting line represents a significant correlation between a pair of biomarkers. Dashed linesrepresent negative correlations. Solid lines represent positive correlations, and the degree of significance is represented by the line thickness [moderate correlation (continuous thin lines) for 0.37<r>0.67 or strong correlation (continuous thick lines) for r>0.68]. Spearman r indexes are used to classify the connecting edges as negative, moderate, or strong positive correlations, as shown.

Aiming to identify the overall balance of the evaluated cytokines, we have applied machine-learning approaches to analyze the cytokine network for all immunization protocols generated upon SLcA and VSA-stimuli *in vitro* (S1 Fig and Fig 4).

The cytokine balance was calculated as the IFN-γ/IL-4 and IFN-γ/IL-10 ratio for all experimental groups and culture conditions and data provide in the S1 Fig. The results re-enforce that ability of “LbSapSal” vaccine to induce a long lasting IFN- production over the synthesis of both IL-4 and IL-10, regardless the antigen-stimuli (S1 Fig).

Data mining by system biology tools were used to generate biomarker networks for each experimental group using both VSA and SLcA-stimuli. The results demonstrated that upon VSA-stimulation, the “Control” group presented negative correlations between IL-4 with TNF-α and IL-10 (VSA stimulus). Positive correlation was also observed between TNF-α and IFN-γ. Upon SLcA-stimulation, positive correlation was observed between IL-12 and IFN-γ along with a negative correlation between TNF-α and IL-4 (Fig 4, upper left panel).

The “Sal” group presented, upon VSA-stimulation, a range of positive correlations, including TNF-α with IL-12, IFN-γ and IL-10 along IL-12 with IL-10. In addition, negative correlation was observed between IFN-γ and IL-4. Analysis upon SLcA-stimulation demonstrated positive correlations amongst the pro-inflammatory cytokines (TNF-α, IFN-γ and IL-12) along positive correlation of them with IL-10 (Fig 4, upper right panel).

Analysis of the “LbSal” group upon VSA-stimulation demonstrated a single negative correlation between TNF-α and IL-4 (Fig 4, bottom left panel).

Data analysis demonstrated that upon VSA-stimulation, the “LbSapSal” group showed strong positive correlation among TNF-α and IFN-γ along with positive correlation between TNF-α and IL-10. Negative correlations were also observed amongst TNF-α and IFN-γ with IL-4 (Fig 4, bottom right panel). Moreover, upon SLcA-stimulation, positive correlation was observed between IL-12 with IFN-γ and IL-10 (Fig 4, bottom right panel).

### A sustained prominent reduction in spleen parasite load was observed in “LbSapSal” group later on after *L*. *chagasi*-challenge

The parasitological analysis performed in spleen samples later on (T_885_) following *L*. *chagasi*-challenge and reported as number of *L*. *chagasi* organisms/20ng of total DNA in spleen samples and presented in Table 1. Data analysis demonstrated that all vaccination protocols were able to induce a reduction in the splenic parasite load as compared to the “Control group”. Indeed, “Sal” and “LbSal” groups yielded 74.6% and 66.5% of reduction in the spleen parasite load (1.56 and 2.06 amastigotes/20ng of total DNA, respectively) as compared to the “Control” group (6.14 amastigotes/20ng of total DNA). “LbSapSal” groups showed the lowest parasite load (1.30 amastigotes/20ng of total DNA), leading to 78.9% of reduction rate in relation to the “Control” group (Table 1). Moreover, no clinical signs and mortality were observed throughout the experimental design (Table 1).

**Table 1:** Parasitological analysis in spleen samples late after (T_885_) *L*. *chagasi*-challenge.

Groups	Dog number	Clinical status	Number of amastigotes per20ng of total DNA
Control	6	Asymptomatic	4.9
	10	Asymptomatic	6.9
	13	Asymptomatic	7.3
	29	Asymptomatic	4.7
	37	Asymptomatic	6.9
**Mean**			**6.14**
Sal	8	Asymptomatic	2.1
	9	Asymptomatic	1.5
	15	Asymptomatic	1.5
	21	Asymptomatic	1.6
	34	Asymptomatic	1.1
**Mean**			**1.56^a^**
**Reduction in parasite load (%)**			**74.6**
LbSal	16	Asymptomatic	2.9
	22	Asymptomatic	1.7
	25	Asymptomatic	1.7
	32	Asymptomatic	1.9
	39	Asymptomatic	2.1
**Mean**			**2.06^a^**
**Reduction in parasite load (%)**			**66.5**
LbSapSal	18	Asymptomatic	1.1
	23	Asymptomatic	1.1
	28	Asymptomatic	1.7
	31	Asymptomatic	1.3
	38	Asymptomatic	1.3
**Mean**			**1.30^a^**
**Reduction in parasite load (%)**			**78.9**

The groups are represented as follows: “Control”; “Sal” (salivary glands of *Lutzomyia longipalpis*); “LbSal” (antigen of *L*. *braziliensis* plus *Lutzomyia longipalpis* salivary glands) and “LbSapSal” (antigen of *L*. *braziliensis* plus saponin and *Lutzomyia longipalpis* salivary glands). The letter “a” indicate significant difference in relation to the Sal, LbSal and LbSapSal groups. Reduction (%) in parasite load was calculated as the proportion of number of amastigotes organisms/20ng of total DNA observed in “Sal”, “LbSal” and “LbSapSal” groups in relation to “Control” group. No clinical signs and mortality were observed throughout the experimental design.

### “LbSapSal” group showed after *L*. *chagasi*-challenge enhanced NO production with negative association with the spleen parasite load

The levels of nitric oxide produced by PBMCs were evaluated upon VSA or SLcA-stimuli *in vitro* early (T_90_) and late (T_885_) after *L*. *chagasi*-challenge.

**Fig 5:**
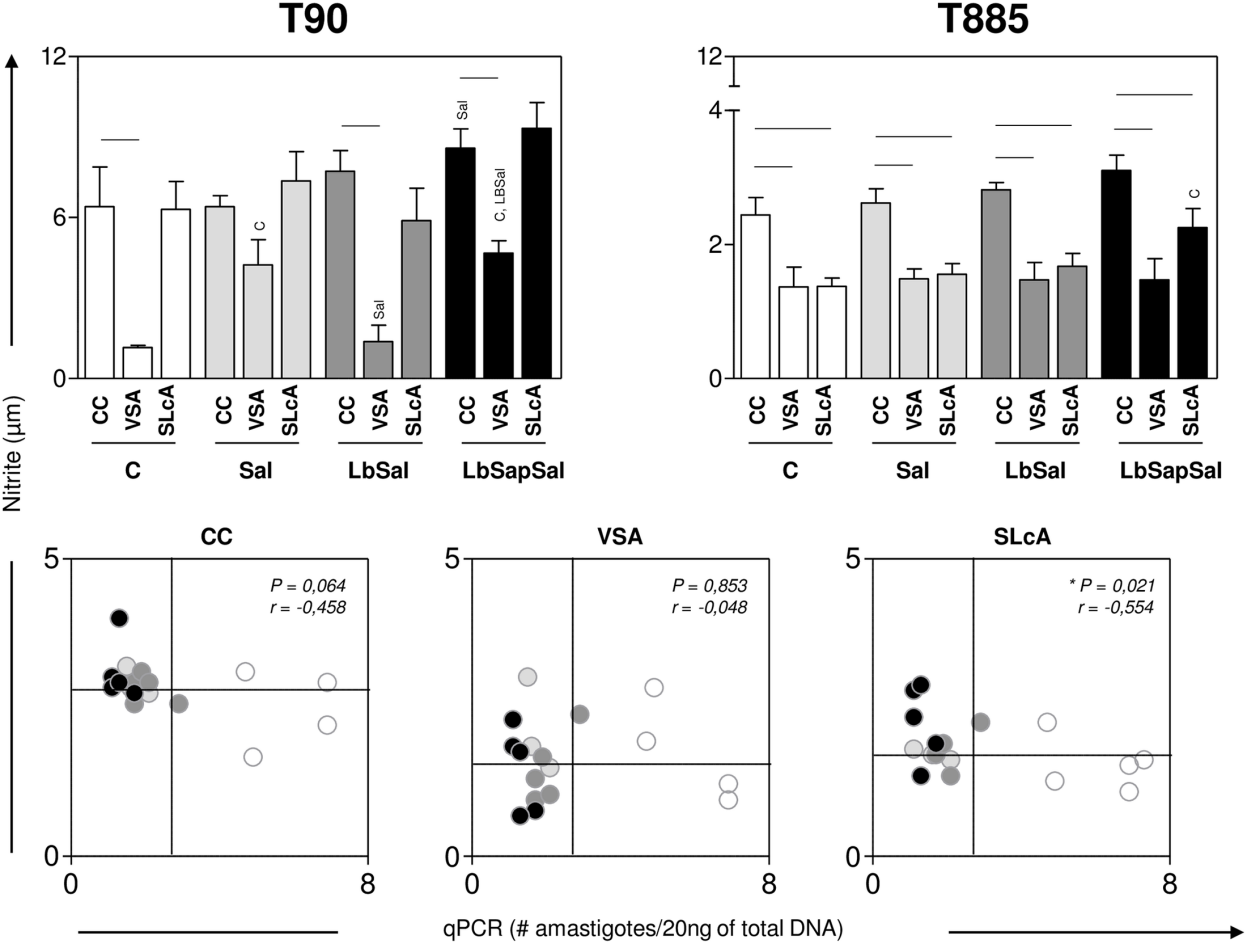
Impact of distinct immunization protocols on the NO production elicited early and late after *L*. *chagasi*-challenge. NO levels (μM) were determined in supernatants from PBMCs cultures maintained upon vaccine-soluble antigen (VSA) or soluble *Leishmania chagasi* antigen (SLcA) stimuli *in vitro*. Data were analyzed early (90 days—T_90_) and late (885 days—T_885_) after experimental *L*. *chagasi*-challenge. The groups are represented as follows: C (“Control”; white bars); “Sal” (*Lutzomyia longipalpis* salivary glands; *light gray bars*); “LbSal” (antigen of *L*. *braziliensis* plus *Lutzomyia longipalpis* salivary glands; *dark gray bars*); and “LbSapSal” (*L*. *braziliensis* antigen plus saponin and *Lutzomyia longipalpis* salivary glands; black bars). Top panels: The x-axis displays the different experimental groups (“Control”, “Sal”, “LbSal” and “LbSapSal”) according to the *in vitro* stimuli (control culture [CC], VSA or SLcA). The y-axis represents the nitrite levels [μM]. Data are presented as mean values ± standard deviations. The connecting lines represent significant difference (*P <0*.*05*) between the CC, VSA or SLcA-stimulated cultures. The symbols C, Sal and LbSal indicate significant differences in comparison to the “Control”, “Sal” or “LbSal” groups, respectively. Bottom panels: Correlation between NO levels and spleen parasite load (# amastigotes/20ng of total DNA) at T_885_ considering CC (bottom left panel) or the presence of a stimulus (VSA: bottom middle panel; or SLcA: bottom right panel) in all groups. The groups are distinguishable by colors as follows: as follows: “C” (white circles); “Sal” (ligh gray circles); “LbSal” (dark gray circles) and “LbSapSal” (black circles). The quadrants represented in the bottom panels delimit the low and high NO producers (y-axis) and the low and high spleen parasite load (x axis).

Data analysis carried early (T_90_) after *L*. *chagasi*-challenge, demonstrated that the “LbSapSal” group presented upon control culture condition enhanced NO levels (*P*<0.05) as compared to the “Sal” group (Fig 5, upper left panel). Additionally, “Sal”, and “LbSapSal” groups showed increase in the NO levels (*P*<0.05) upon VSA-stimulation as compared to the “Control” and “LbSap” groups (Fig 5, upper left panel).

The analysis performed late (T_885_) after *L*. *chagasi*-challenge showed the “LbSapSal” group displayed higher NO levels (*P*<0.05) upon SLcA-stimulation as compared to the “Control” group (Fig 5, upper right panel).

Additional analysis revealed that the NO production at T_885_ displayed a negative correlation with the spleen parasite load selectively upon SLcA-stimulation (Fig 5, bottom right panel). Interestingly, it was observed that in the “LbSapSal” group, four out of five animals (80%) showed simultaneous high NO production and low spleen parasite load, compatible with a protection profile (Fig 5, bottom right panel). In contrast, in the “Control” group, only one out of five (20%) showed high NO production but all animals presented high spleen parasitism (Fig 5, bottom right panel).

## Discussion

The control of *Leishmania chagasi* (syn. *Leishmania infantum*) infection in dogs is essential to stop the current spread of zoonotic visceral leishmaniasis. Therefore, a vaccine against VL would be an important tool for controlling CVL and would dramatically decrease anxiety regarding *L*. *chagasi* infection in humans [18,47]. In this sense, the establishment of biomarkers of immunogenicity is considered critical in the rational approach for analyzing candidate vaccines against CVL, and it contributes to identifying the pattern of immune response in dogs and the search for vaccine candidates against CVL [48,49]. For this reason, in this work, the immunogenicity and protective effects of the “LbSapSal” vaccination in dogs were investigated using levels of NO and cytokines and evaluations related to the spleen parasite load. Furthermore, the addition of sand fly saliva extract to vector-based vaccines can enhance the ability of the host to control or block *Leishmania* infection [38,33].

The major results observed after “LbSapSal” immunization revealed a reduction in IL-4 levels during the early (T_90_) post-challenge period. Importantly, previous studies have identified that the presence of IL-4 in splenocytes from dogs naturally infected with *L*. *chagasi* would be an important biomarker during ongoing CVL [12,50]. However, in some reports, the role of IL-4 was related to the resistance profile or susceptibility profile of CVL [50,51]; the higher levels of IL-4 are considered the hallmark of dogs naturally infected with *L*. *chagasi* [52] and sustained reduction of IL-4 has been also reported by other immunogen candidates. In fact, reduction in the levels of IL-4 after vaccine immunization against CVL has been considered a biomarker for protection against *Leishmania* infection [53].

In the present study, the evaluation of IL-10 demonstrated a possible association with events related to the susceptibility to infection by *L*. *chagasi*; the “Control” group has presented increased amounts during the late (T_885_) post-challenge period. In fact, this cytokine has been associated with severity of VL [54] and CVL [12,16,50,52,55,56]. Importantly, the “LbSapSal” group did not have increased IL-10 production, even after the late *L*. *chagasi-*challenge period.

Additionally, analysis of TGF-β revealed reduced production in the “LbSapSal”-immunized group during the early and late post-challenge periods. Interestingly, the presence of TGF-β has been associated with inducing immunosuppression characteristics during the course of VL [57,58]. Moreover, the presence of TGF-β *in vitro* has a protective effect for amastigotes in macrophages, favoring the maintenance of parasitism [59]. In addition, Alves et al. [50] reported high levels of TGF-β associated with increased parasite load in lymph nodes from symptomatic dogs and concluded that TGF-β is associated with morbidity in CVL. In this context, it is possible to hypothesize that lower levels of TGF-β in the “LbSapSal” group would indicate the establishment of immunoprotective mechanisms, whereas higher amounts in the “Control” group would be associated with the susceptibility pattern after *L*. *chagasi*-challenge.

Analysis of pro-inflammatory cytokines has been considered a prerequisite for composing immunogenicity analyses before and after experimental challenge with *L*. *chagasi* in clinical trials of anti-CVL vaccines [48,26]. The analysis of TNF-α before vaccine immunization (T_0_) demonstrated increased levels in SLcA-stimulated cultures in the “Control” and “LbSapSal” groups and demonstrated a tendency for enhanced amounts in the “Sal” and “LbSal” groups. Similar results were observed for IL-12 and IFN-γ at T_0_. These results seem to indicate that SLcA stimulation would induce increased levels of these cytokines, pointing to an inherent feature of the antigenic stimulus. For this reason, we evaluated all groups and analyzed the same stimulus in each group to identify a cytokine profile regarding type I immune response. This strategy demonstrated enhanced levels of IL-12 and IFN-γ post-vaccination and sustained production of both TNF-α and IFN-γ even after early and late post-challenge periods in the “LbSapSal” group. Some studies have described TNF-α as being associated with a resistance profile in CVL [52,60, 61,62,63] or in dogs vaccinated against CVL [53], and has been associated with susceptibility when associated with high levels of IL-4 and IL-10 [64]. Results obtained by Strauss-Ayali et al. [65] showed that after stimulation with exogenous IL-12, PBMCs from *L*. *infantum*−infected dogs were able to reverse an apparent state of anergy, resulting in increased production of IFN-γ. Moreover, Menezes-Souza et al. [62] found that low levels of IL-12 concomitant with high levels of IL-10 and TGF-β represent a favorable condition for the persistence and replication of parasites in CVL. It has been described that IFN-γ is linked to a resistance profile in different experimental models for VL [50,61,66,67,68] and in dogs vaccinated against CVL [21,26,29, 30,53].

Our data regarding the cytokine network indicated a balanced immune response in the “LbSapSal” group. In this sense, we describe positive (TNF-α versus IFN-γ and IL-10; IL-12 versus IFN-γ and IL-10) and negative (IL-4 versus TNF-α or IFN-γ) correlations demonstrating a prominent pro-inflammatory immune response in the “LbSapSal” group. In addition to a non-significant increase in IL-4 or IL-10 levels in the “LbSapSal” group, a balanced immune response was demonstrated by taking into account the positive correlations between IL-10 and type I cytokines (IFN-γ, IL-12, TNF-α). These data should be related to the regulation of the prominent pro-inflammatory immune response induced by the “LbSapSal” vaccination and should aim to control potential tissue damage by type I cytokines.

The parasitological evaluation revealed in “LbSapSal” group a remarkable reduction in spleen parasite load (78.9%) after experimental *L*. *chagasi*-challenge, in concordance with a prominent pro-inflammatory immune response induced by this vaccine. In fact, we have been described a parasite load reduction of 69% in the LBSapSal group [27], indicating the capacity of this vaccine to control parasite replication even long after challenge (885 days).

Sand fly saliva displays an important role in the first steps of *Leishmania* infection, due to the vast repertoire of pharmacologically active molecules that surround host’s hemostatic system [69]. The re-exposure to the salivary components seems to display an immunogenic activity, eliciting antibody production and cell mediated immunity by the host that could be block or limit the *Leishmania* infection [70]. Some studies have been shown that *L*. *longipalpis* salivary proteins induce an immune response associated with protection in dogs [22,27]. Furthermore, in a hamster model, salivary proteins of a sand fly protects against the fatal outcome of visceral leishmaniasis [40]. Similarly, we observed 74.6% parasite load reduction in “Sal” group, showing that salivary components have a high potential to limit infection in dogs.

The experimental challenge in vaccine studies against canine visceral leishmaniasis is considered as crucial to analyze the protection performance. Distinct studies have been published aiming to determine the experimental *L*. *chagasi-*challenge plus sand fly saliva using intradermal route in dogs would be more similar to natural infection than intravenous challenge [71,72]. However, using intradermal challenge, the dogs would be asymptomatic during all the study, besides to present lower parasitism [71]. Since the “LbSapSal” vaccine presents saliva as antigenic compound, the ideal experimental challenge to test the protection should ideally be performed by intradermal route, as analyzed in our study.

Importantly, we observed increased NO levels in the “LbSapSal” group during the early and late post-challenge periods. Interestingly, four out of five dogs immunized with “LbSapSal” presented higher NO amounts and low spleen parasite burden during the late post-challenge period, indicating long-term immunogenicity and resulting in reduction of parasitism. In fact, we have previously demonstrated that “LbSapSal” induced resistance biomarkers specifically related to expansion of circulating CD4^+^ and CD8^+^ T-cells and *Leishmania*-specific subsets and lower levels of parasitism [22,27]. Panaro et al. [73] also observed an increase in NO production and anti-leishmanial activity of macrophages, as well as increased levels of IFN-γ in PBMCs supernatants, in dogs immunized with a vaccine comprising crude antigens of *L*. *infantum*.

Taken together, our major data indicate that immunization with “LbSapSal” is able to induce a protection profile characterized by enhanced amounts of type I (TNF-α, IL-12, IFN-γ) cytokines and reduction in type II cytokines (IL-4 and TGF-β), even after experimental challenge. The establishment of a polarized type I immune response after “LbSapSal” immunization supported increased levels of NO production, favoring a reduction in parasitism and indicating long-lasting protection against *L*. *chagasi* infection. These results encourage further studies that can provide important information for a better understanding of the effectiveness of the “LbSapSal” vaccine and strategies for addressing *Leishmania* antigens in combination with sand fly proteins such as those present in the saliva in the vector.

## Supporting Information

S1 Fig Impact of distinct immunization protocols on the pro-inflammatory/regulatory cytokine balance. The balance of inflammatory cytokine IFN-γ and regulatory/anti-inflammatory (IL-4 and IL-10) were analyzed in the supernatant of PBMCs maintained upon vaccine-soluble antigen (VSA) or soluble *Leishmania chagasi* antigen (SLcA) stimuli *in vitro*. Data were analyzed early (90 days—T_90_) and late (885 days—T_885_) after experimental *L*. *chagasi*-challenge. The groups are represented as follows: C (“Control”; white bars); “Sal” (*Lutzomyia longipalpis* salivary glands; *light gray bars*); “LbSal” (antigen of *L*. *braziliensis* plus *Lutzomyia longipalpis* salivary glands; *dark gray bars*); and “LbSapSal” (*L*. *braziliensis* antigen plus saponin and *Lutzomyia longipalpis* salivary glands; black bars).The x-axis displays the different experimental groups (“Control”, “Sal”, “LbSal” and “LbSapSal”) according to the *in vitro* stimuli (control culture [CC], VSA or SLcA). The y-axis represents the cytokine ratio (IFN-γ/IL4 and IFN-γ/IL-10). Data are presented as mean values ± standard deviations. The connecting lines represent significant difference (*P <0*.*05*) amongst the CC, VSA or SLcA-stimulated cultures. The symbols C and Sal indicate significant differences in comparison to the “Control” or “Sal” groups, respectively.
(TIF)
