# Supplementary figures and images for: Impact of LbSapSal Vaccine in Canine Immunological and Parasitological Features before and after Leishmania chagasi-Challenge
Source: PLoS One. 2016 Aug 24;11(8):e0161169. doi: 10.1371/journal.pone.0161169 (PMC4996460; doi:10.1371/journal.pone.0161169)

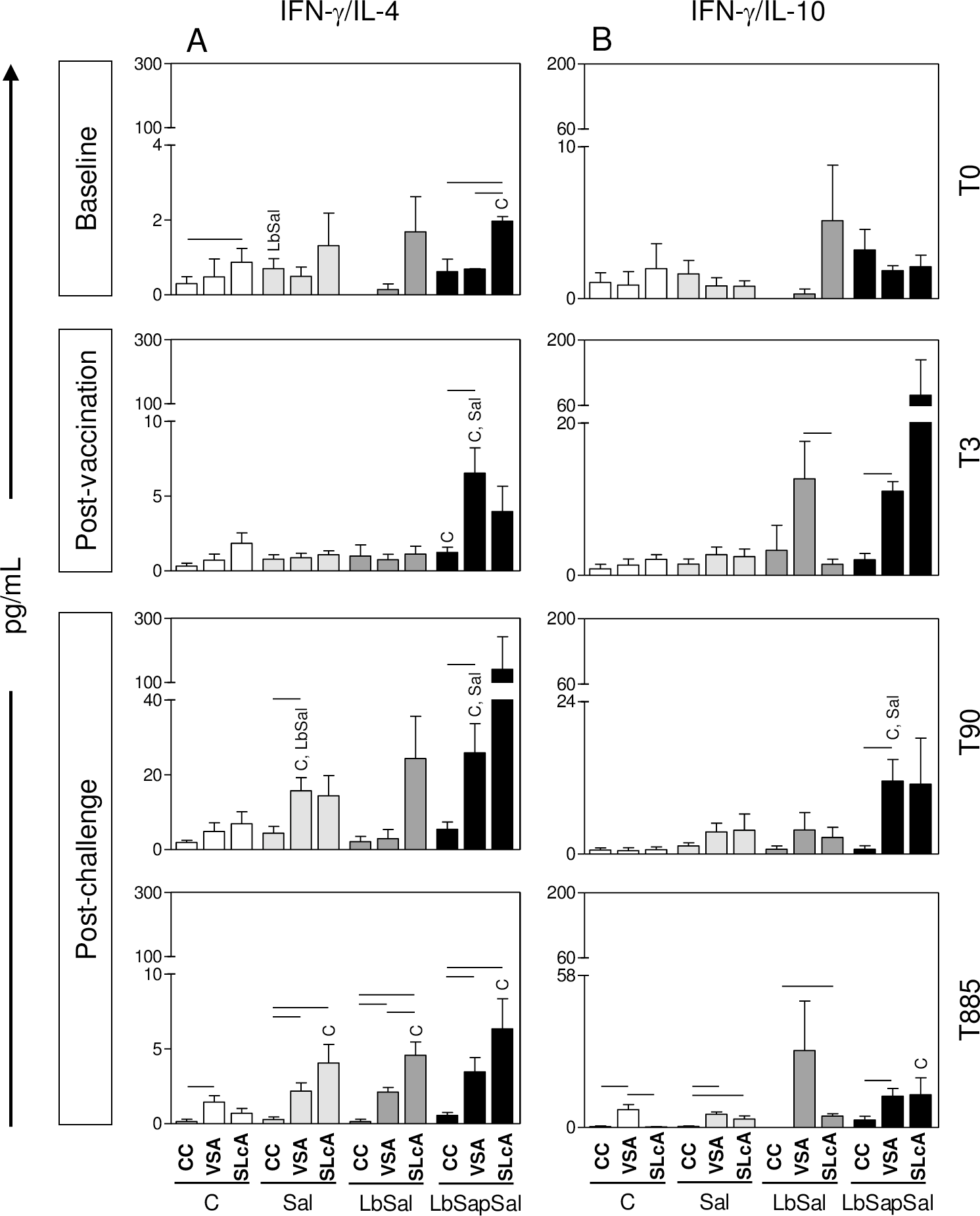

Supplement: S1 Fig — The balance of inflammatory cytokine IFN-γ and regulatory/anti-inflammatory (IL-4 and IL-10) were analyzed in the supernatant of PBMCs maintained upon vaccine-soluble antigen (VSA) or soluble Leishmania chagasi antigen (SLcA) stimuli in vitro. Data were analyzed early (90 days—T90) and late (885 days—T885) after experimental L. chagasi-challenge. The groups are represented as follows: C (“Control”; white bars); “Sal” (Lutzomyia longipalpis salivary glands; light gray bars); “LbSal” (antigen of L. braziliensis plus Lutzomyia longipalpis salivary glands; dark gray bars); and “LbSapSal” (L. braziliensis antigen plus saponin and Lutzomyia longipalpis salivary glands; black bars).The x-axis displays the different experimental groups (“Control”, “Sal”, “LbSal” and “LbSapSal”) according to the in vitro stimuli (control culture [CC], VSA or SLcA). The y-axis represents the cytokine ratio (IFN-γ/IL4 and IFN-γ/IL-10). Data are presented as mean values ± standard deviations. The connecting lines represent significant difference (P <0.05) amongst the CC, VSA or SLcA-stimulated cultures. The symbols C and Sal indicate significant differences in comparison to the “Control” or “Sal” groups, respectively. (TIF) [file pone.0161169.s001.tif]
